# Letter to the Editor regarding “Long-term sustainability of improvements in antibiotic prescribing after implementation of a local guideline for the management of patients hospitalized with skin and soft tissue infection” by Frappa *et al*.

**DOI:** 10.1017/ash.2025.10116

**Published:** 2025-09-05

**Authors:** Callan Bleick

**Affiliations:** Anti-Infective Research Laboratory, Department of Pharmacy Practice, Eugene Applebaum College of Pharmacy and Health Sciences, Wayne State University, Detroit, MI, USA

Dear Editor,

I have read with great interest the article, “Long-Term Sustainability of Improvements in Antibiotic Prescribing after Implementation of a Local Guideline for the Management of Patients Hospitalized with Skin and Soft Tissue Infection” to be published in Antimicrobial Stewardship and Healthcare Epidemiology Journal by Frappa *et al*. This retrospective, single-center study implements a common approach often used in real-world antimicrobial stewardship scenarios, providing pragmatic insights that can inform clinical practice and guide stewardship initiatives at institutions alike.

I agree with the authors’ remarks that skin and soft tissue infections (SSTIs) are among the most frequently treated infections in institutions, where antibiotic overuse remains a concern.^1–3^ This issue is further compounded by prolonged treatments, frequent use of broad-spectrum agents, and rising antimicrobial resistance trends.^4–6^ The authors effectively reinforce prior evidence demonstrating that implementing local guidelines improves antimicrobial stewardship efforts, serving as a practical, evidence-based intervention with a proven track record of success.^7–9^

This retrospective study evaluated changes in antibiotic use at Denver Health Medical Center by comparing three distinct time periods: preintervention, during guideline implementation, and postintervention maintenance for patients hospitalized with skin and soft tissue infections, specifically cellulitis and cutaneous abscesses. The authors reviewed patient records using ICD-10 codes and manual chart review to identify eligible cases applying exclusion criteria as appropriate. Primary and co-primary outcomes as well as trends between time periods were analyzed via interrupted time series (ITS) regression models with autoregressive moving average (ARMA) adjustments, along with the Kruskal-Wallis test. Future studies with access to a larger patient database or multi-centered cohorts could consider applying advanced statistical methods such as propensity score matching (PSM) to control for cofounding^10,11^ and segmented mixed-effects models to account for both patient-level and time-related variability.^12^ Additionally, incorporating difference-in-differences (DiD) analysis if a comparable control group becomes available could be beneficial^13^ and Bayesian ITS models could further quantify uncertainty around intervention impact.^14^

The authors describe key findings such as a substantial decline in broad-spectrum antibiotic use following guideline implementation, decreasing from 67% before implementation to 37% during, and 27% in the subsequent years. Antibiotic use for antipseudomonal coverage also declined from 28% to 16%. Similarly, the median duration of antibiotic therapy decreased from approximately 13 days to 8 days over time. Most importantly, these reductions and antibiotic use did not negatively impact patient outcomes as the authors concluded there were no significant differences in hospital length of stay, hospital mortality, or 30-day readmission. The summary of the results highlight that operationalizing local guidelines can effectively minimize unnecessary antibiotic use without compromising patient care.

I found this study particularly interesting as it assessed the impact of institutional guidelines for skin and soft tissue infections, specifically related to cellulitis and cutaneous abscesses. Despite the acknowledged limitations by the authors, including the data gap and small quarterly sample sizes, the consistency of trends the authors provide during the maintenance period underscores the durability and effectiveness of guideline-driven interventions. Expanding future work to include other SSTIs could be valuable; however, I believe the strength of this study lies in its long-term evaluation of prescribing habits and changes over time in relation to patient outcomes following the guideline implementation period. This research is important and needed as it reinforces prior evidence demonstrating the positive impact of institution-specific guideline implementation on both patient care and hospital practice, especially in resource-limited settings. In addition, an often-underreported aspect in antimicrobial stewardship literature is the long-term sustainability of practice changes without active oversight, which I believe the authors uniquely address and demonstrate through their extended follow-up period.

Multi-center studies can offer broad generalizability; however, the depth of this single-center, real-world study provides meaningful insight into what sustained success can look like within a healthcare system like Denver Health Medical Center. I would encourage future research to explore patient and prescriber factors contributing to sustained adherence and to evaluate whether similar success can be replicated in other health conditions as well as in larger- or smaller-scale institutions. In general, the article by Frappa *et al.* provides timely practical evidence supporting the prioritization of guideline implementation in stewardship programs. The findings discussed will be of particular interest to stewardship practitioners advocating for sustainable, low-resource interventions that influence prescribing culture long term.
